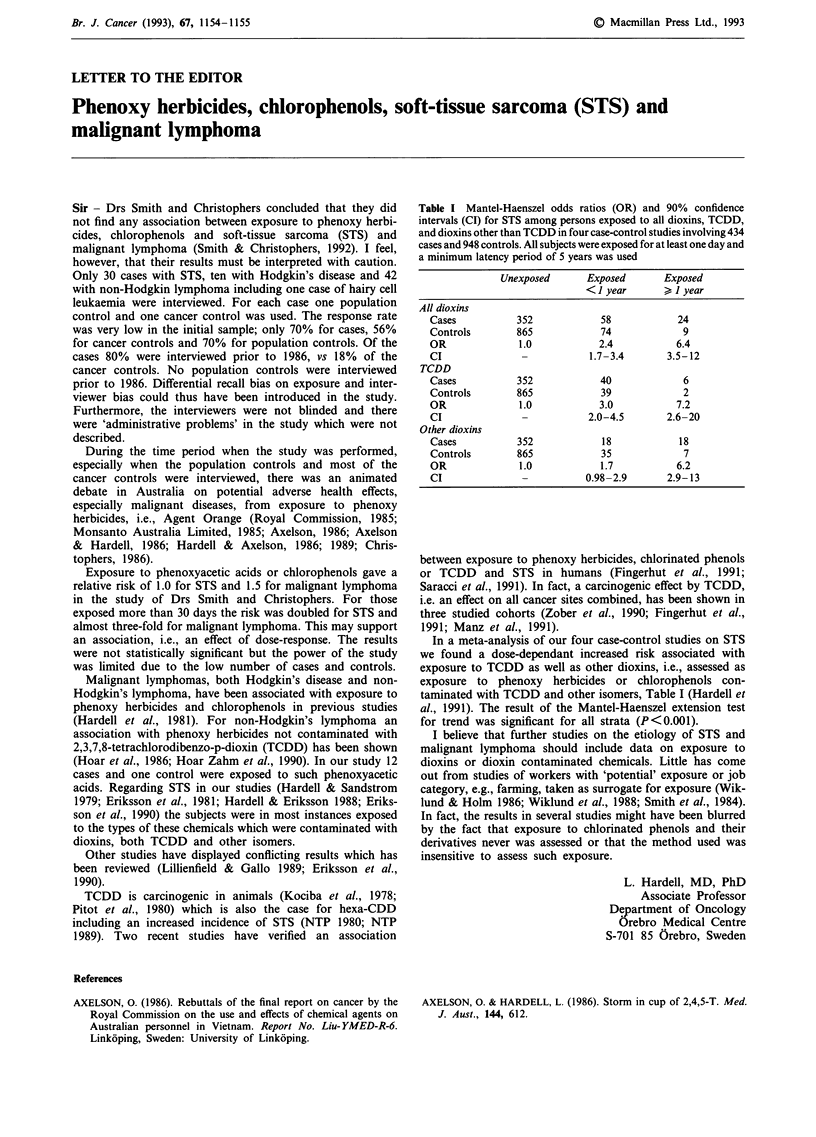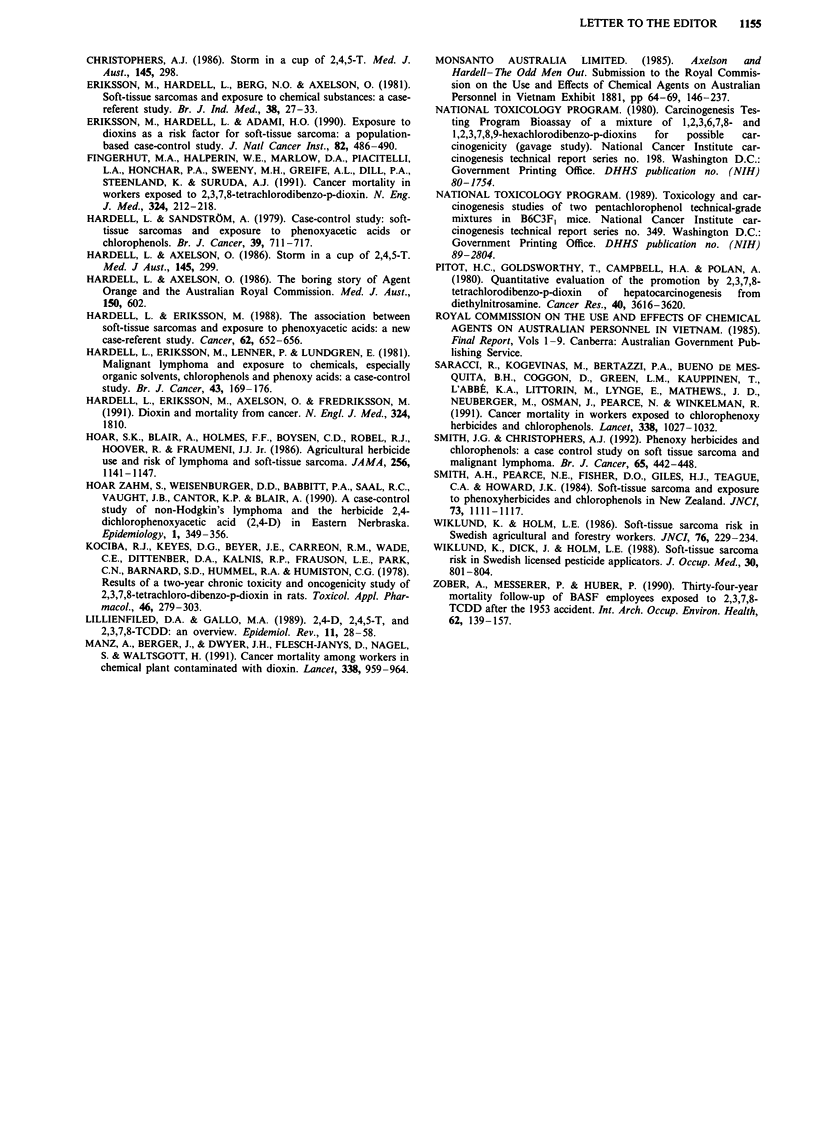# Phenoxy herbicides, chlorophenols, soft-tissue sarcoma (STS) and malignant lymphoma.

**DOI:** 10.1038/bjc.1993.212

**Published:** 1993-05

**Authors:** L. Hardell


Br. J. Cancer (1993), 67, 1154–1155 © Macmillan Press Ltd., 1993

LETTER TO THE EDITOR

### Phenoxy herbicides, chlorophenols, soft-tissue sarcoma (STS) and malignant lymphoma

**Sir** – Drs Smith and Christophers concluded that they did not find any association between exposure to phenoxy herbicides, chlorophenols and soft-tissue sarcoma (STS) and malignant lymphoma (Smith & Christophers, 1992). I feel, however, that their results must be interpreted with caution. Only 30 cases with STS, ten with Hodgkin's disease and 42 with non-Hodgkin lymphoma including one case of hairy cell leukaemia were interviewed. For each case one population control and one cancer control was used. The response rate was very low in the initial sample; only 70% for cases, 56% for cancer controls and 70% for population controls. Of the cases 80% were interviewed prior to 1986, *vs* 18% of the cancer controls. No population controls were interviewed prior to 1986. Differential recall bias on exposure and interviewer bias could thus have been introduced in the study. Furthermore, the interviewers were not blinded and there were 'administrative problems' in the study which were not described.

During the time period when the study was performed, especially when the population controls and most of the cancer controls were interviewed, there was an animated debate in Australia on potential adverse health effects, especially malignant diseases, from exposure to phenoxy herbicides, i.e., Agent Orange (Royal Commission, 1985; Monsanto Australia Limited, 1985; Axelson, 1986; Axelson & Hardell, 1986; Hardell & Axelson, 1986; 1989; Christophers, 1986).

Exposure to phenoxyacetic acids or chlorophenols gave a relative risk of 1.0 for STS and 1.5 for malignant lymphoma in the study of Drs Smith and Christophers. For those exposed more than 30 days the risk was doubled for STS and almost three-fold for malignant lymphoma. This may support an association, i.e., an effect of dose-response. The results were not statistically significant but the power of the study was limited due to the low number of cases and controls.

Malignant lymphomas, both Hodgkin's disease and non-Hodgkin's lymphoma, have been associated with exposure to phenoxy herbicides and chlorophenols in previous studies (Hardell *et al.*, 1981). For non-Hodgkin's lymphoma an association with phenoxy herbicides not contaminated with 2,3,7,8-tetrachlorodibenzo-p-dioxin (TCDD) has been shown (Hoar *et al.*, 1986; Hoar Zahm *et al.*, 1990). In our study 12 cases and one control were exposed to such phenoxyacetic acids. Regarding STS in our studies (Hardell & Sandstrom 1979; Eriksson *et al.*, 1981; Hardell & Eriksson 1988; Eriksson *et al.*, 1990) the subjects were in most instances exposed to the types of these chemicals which were contaminated with dioxins, both TCDD and other isomers.

Other studies have displayed conflicting results which has been reviewed (Lillienfield & Gallo 1989; Eriksson *et al.*, 1990).

TCDD is carcinogenic in animals (Kociba *et al.*, 1978; Pitot *et al.*, 1980) which is also the case for hexa-CDD including an increased incidence of STS (NTP 1980; NTP 1989). Two recent studies have verified an association between exposure to phenoxy herbicides, chlorinated phenols or TCDD and STS in humans (Fingerhut *et al.*, 1991; Saracci *et al.*, 1991). In fact, a carcinogenic effect by TCDD, i.e. an effect on all cancer sites combined, has been shown in three studied cohorts (Zober *et al.*, 1990; Fingerhut *et al.*, 1991; Manz *et al.*, 1991).

**Table I** Mantel-Haenszel odds ratios (OR) and 90% confidence intervals (CI) for STS among persons exposed to all dioxins, TCDD, and dioxins other than TCDD in four case-control studies involving 434 cases and 948 controls. All subjects were exposed for at least one day and a minimum latency period of 5 years was used

| | *Unexposed* | *Exposed* $<1$ *year* | *Exposed* $\geq 1$ *year* |
|---|---|---|---|
| *All dioxins* | | | |
| Cases | 352 | 58 | 24 |
| Controls | 865 | 74 | 9 |
| OR | 1.0 | 2.4 | 6.4 |
| CI | – | 1.7–3.4 | 3.5–12 |
| *TCDD* | | | |
| Cases | 352 | 40 | 6 |
| Controls | 865 | 39 | 2 |
| OR | 1.0 | 3.0 | 7.2 |
| CI | – | 2.0–4.5 | 2.6–20 |
| *Other dioxins* | | | |
| Cases | 352 | 18 | 18 |
| Controls | 865 | 35 | 7 |
| OR | 1.0 | 1.7 | 6.2 |
| CI | – | 0.98–2.9 | 2.9–13 |

In a meta-analysis of our four case-control studies on STS we found a dose-dependant increased risk associated with exposure to TCDD as well as other dioxins, i.e., assessed as exposure to phenoxy herbicides or chlorophenols contaminated with TCDD and other isomers, Table I (Hardell *et al.*, 1991). The result of the Mantel-Haenszel extension test for trend was significant for all strata ($P<0.001$).

I believe that further studies on the etiology of STS and malignant lymphoma should include data on exposure to dioxins or dioxin contaminated chemicals. Little has come out from studies of workers with 'potential' exposure or job category, e.g., farming, taken as surrogate for exposure (Wiklund & Holm 1986; Wiklund *et al.*, 1988; Smith *et al.*, 1984). In fact, the results in several studies might have been blurred by the fact that exposure to chlorinated phenols and their derivatives never was assessed or that the method used was insensitive to assess such exposure.

L. Hardell, MD, PhD
Associate Professor
Department of Oncology
Örebro Medical Centre
S-701 85 Örebro, Sweden

**References**

AXELSON, O. (1986). Rebuttals of the final report on cancer by the Royal Commission on the use and effects of chemical agents on Australian personnel in Vietnam. *Report No. Liu-YMED-R-6*. Linköping, Sweden: University of Linköping.

AXELSON, O. & HARDELL, L. (1986). Storm in cup of 2,4,5-T. *Med. J. Aust.*, **144**, 612.

CHRISTOPHERS, A.J. (1986). Storm in a cup of 2,4,5-T. *Med. J. Aust.*, **145**, 298.

ERIKSSON, M., HARDELL, L., BERG, N.O. & AXELSON, O. (1981). Soft-tissue sarcomas and exposure to chemical substances: a case-referent study. *Br. J. Ind. Med.*, **38**, 27–33.

ERIKSSON, M., HARDELL, L. & ADAMI, H.O. (1990). Exposure to dioxins as a risk factor for soft-tissue sarcoma: a population-based case-control study. *J. Natl Cancer Inst.*, **82**, 486–490.

FINGERHUT, M.A., HALPERIN, W.E., MARLOW, D.A., PIACITELLI, L.A., HONCHAR, P.A., SWEENY, M.H., GREIFE, A.L., DILL, P.A., STEENLAND, K. & SURUDA, A.J. (1991). Cancer mortality in workers exposed to 2,3,7,8-tetrachlorodibenzo-p-dioxin. *N. Eng. J. Med.*, **324**, 212–218.

HARDELL, L. & SANDSTRÖM, A. (1979). Case-control study: soft-tissue sarcomas and exposure to phenoxyacetic acids or chlorophenols. *Br. J. Cancer*, **39**, 711–717.

HARDELL, L. & AXELSON, O. (1986). Storm in a cup of 2,4,5-T. *Med. J Aust.*, **145**, 299.

HARDELL, L. & AXELSON, O. (1986). The boring story of Agent Orange and the Australian Royal Commission. *Med. J. Aust.*, **150**, 602.

HARDELL, L. & ERIKSSON, M. (1988). The association between soft-tissue sarcomas and exposure to phenoxyacetic acids: a new case-referent study. *Cancer*, **62**, 652–656.

HARDELL, L., ERIKSSON, M., LENNER, P. & LUNDGREN, E. (1981). Malignant lymphoma and exposure to chemicals, especially organic solvents, chlorophenols and phenoxy acids: a case-control study. *Br. J. Cancer*, **43**, 169–176.

HARDELL, L., ERIKSSON, M., AXELSON, O. & FREDRIKSSON, M. (1991). Dioxin and mortality from cancer. *N. Engl. J. Med.*, **324**, 1810.

HOAR, S.K., BLAIR, A., HOLMES, F.F., BOYSEN, C.D., ROBEL, R.J., HOOVER, R. & FRAUMENI, J.J. Jr. (1986). Agricultural herbicide use and risk of lymphoma and soft-tissue sarcoma. *JAMA*, **256**, 1141–1147.

HOAR ZAHM, S., WEISENBURGER, D.D., BABBITT, P.A., SAAL, R.C., VAUGHT, J.B., CANTOR, K.P. & BLAIR, A. (1990). A case-control study of non-Hodgkin's lymphoma and the herbicide 2,4-dichlorophenoxyacetic acid (2,4-D) in Eastern Nerbraska. *Epidemiology*, **1**, 349–356.

KOCIBA, R.J., KEYES, D.G., BEYER, J.E., CARREON, R.M., WADE, C.E., DITTENBER, D.A., KALNIS, R.P., FRAUSON, L.E., PARK, C.N., BARNARD, S.D., HUMMEL, R.A. & HUMISTON, C.G. (1978). Results of a two-year chronic toxicity and oncogenicity study of 2,3,7,8-tetrachloro-dibenzo-p-dioxin in rats. *Toxicol. Appl. Pharmacol.*, **46**, 279–303.

LILLIENFILED, D.A. & GALLO, M.A. (1989). 2,4-D, 2,4,5-T, and 2,3,7,8-TCDD: an overview. *Epidemiol. Rev.*, **11**, 28–58.

MANZ, A., BERGER, J., & DWYER, J.H., FLESCH-JANYS, D., NAGEL, S. & WALTSGOTT, H. (1991). Cancer mortality among workers in chemical plant contaminated with dioxin. *Lancet*, **338**, 959–964.

MONSANTO AUSTRALIA LIMITED. (1985). *Axelson and Hardell–The Odd Men Out*. Submission to the Royal Commission on the Use and Effects of Chemical Agents on Australian Personnel in Vietnam Exhibit 1881, pp 64–69, 146–237.

NATIONAL TOXICOLOGY PROGRAM. (1980). Carcinogenesis Testing Program Bioassay of a mixture of 1,2,3,6,7,8- and 1,2,3,7,8,9-hexachlorodibenzo-p-dioxins for possible carcinogenicity (gavage study). National Cancer Institute carcinogenesis technical report series no. 198. Washington D.C.: Government Printing Office. *DHHS publication no. (NIH) 80–1754.*

NATIONAL TOXICOLOGY PROGRAM. (1989). Toxicology and carcinogenesis studies of two pentachlorophenol technical-grade mixtures in $B6C3F_1$ mice. National Cancer Institute carcinogenesis technical report series no. 349. Washington D.C.: Government Printing Office. *DHHS publication no. (NIH) 89–2804.*

PITOT, H.C., GOLDSWORTHY, T., CAMPBELL, H.A. & POLAN, A. (1980). Quantitative evaluation of the promotion by 2,3,7,8-tetrachlorodibenzo-p-dioxin of hepatocarcinogenesis from diethylnitrosamine. *Cancer Res.*, **40**, 3616–3620.

ROYAL COMMISSION ON THE USE AND EFFECTS OF CHEMICAL AGENTS ON AUSTRALIAN PERSONNEL IN VIETNAM. (1985). *Final Report*, Vols 1–9. Canberra: Australian Government Publishing Service.

SARACCI, R., KOGEVINAS, M., BERTAZZI, P.A., BUENO DE MESQUITA, B.H., COGGON, D., GREEN, L.M., KAUPPINEN, T., L'ABBÉ, K.A., LITTORIN, M., LYNGE, E., MATHEWS., J. D., NEUBERGER, M., OSMAN, J., PEARCE, N. & WINKELMAN, R. (1991). Cancer mortality in workers exposed to chlorophenoxy herbicides and chlorophenols. *Lancet*, **338**, 1027–1032.

SMITH, J.G. & CHRISTOPHERS, A.J. (1992). Phenoxy herbicides and chlorophenols: a case control study on soft tissue sarcoma and malignant lymphoma. *Br. J. Cancer*, **65**, 442–448.

SMITH, A.H., PEARCE, N.E., FISHER, D.O., GILES, H.J., TEAGUE, C.A. & HOWARD, J.K. (1984). Soft-tissue sarcoma and exposure to phenoxyherbicides and chlorophenols in New Zealand. *JNCI*, **73**, 1111–1117.

WIKLUND, K. & HOLM, L.E. (1986). Soft-tissue sarcoma risk in Swedish agricultural and forestry workers. *JNCI*, **76**, 229–234.

WIKLUND, K., DICK, J. & HOLM, L.E. (1988). Soft-tissue sarcoma risk in Swedish licensed pesticide applicators. *J. Occup. Med.*, **30**, 801–804.

ZOBER, A., MESSERER, P. & HUBER, P. (1990). Thirty-four-year mortality follow-up of BASF employees exposed to 2,3,7,8-TCDD after the 1953 accident. *Int. Arch. Occup. Environ. Health*, **62**, 139–157.